# Supplementary material for: The role of operators in sustainable whale-watching tourism: Proposing a continuous training framework
Source: PLoS One. 2024 Jan 2;19(1):e0296241. doi: 10.1371/journal.pone.0296241 (PMC10760867; doi:10.1371/journal.pone.0296241)
Supplement: S1 Table — Questionnaire for whale-watching operators used in this paper. (PDF) [file pone.0296241.s001.pdf]

S1 Table: Questionnaire for whale-watching operators used in this paper:

**Trip specific questions**<br>Your answers will help us understand more about your work as a whale-watch operator.	
Which routes do you usually take? Choose a number corresponding to the area on the map (Image modified from Google Earth). Select all that might apply.	
How far away does your vessel go from the whale during the tours?	• More than 100 m<br>• More than 200 m<br>• Less than 10 m<br>• 10 - 50 m<br>• Around 100 m<br>• I don't know
Do you think that there are any issues related to the interaction between ships and marine fauna? Select all that might apply.	• Chemical Pollution<br>• Ship strikes<br>• Noise<br>• Introduction of invasive species<br>• Biofouling<br>• I don't know<br>• None of them<br>• All of them
**Underwater noise**<br>Your answers will help us understand more about your relation with cetaceans as a whale-watching tour operator. In particular, we are interested in the acoustic interaction between whale-watching activity and cetaceans.	
What are the most important aspects for tourists? Select all that might apply.	• Going very close to the whales<br>• Being taught something about the biology/ecology of the whales<br>• Seeing as many animals as possible<br>• Getting to know something about the marine environment of the area<br>• Other<br>• Seeing at least one whale during the trip<br>• I don't know
How far away should the vessel be from the cetacean, at least?	• 100 m<br>• 200 m<br>• 300 m<br>• 350 m<br>• I don't know<br>• It doesn't matter

Which of these strategies would you prefer to follow in order to reduce the impact of underwater noise from shipping on cetaceans? (Select all that might apply)	• Implement mandatory AIS on all vessels (even small recreational ones)<br>• Increase distance from the whales<br>• Decrease the speed of the vessel near whale migratory routes or selected areas<br>• Increase the number of Marine Protected Areas with limited access for vessels with a permit<br>• Implement mandatory avoidance of feeding and breeding areas during the most important times of day for these activities<br>• Diminish the duration of the whale-watching trips<br>• Avoid whale-watching tours during specific times of day<br>• Increase the duration of the whale-watching trips, but reduce the trips to one, daily
