# Supplementary material for: The role of operators in sustainable whale-watching tourism: Proposing a continuous training framework
Source: PLoS One. 2024 Jan 2;19(1):e0296241. doi: 10.1371/journal.pone.0296241 (PMC10760867; doi:10.1371/journal.pone.0296241)
Supplement: S2 Table — Whale-watching experts’ questionnaire. Questions between both questionnaires are linked by their identifier (ID). Questions marked with an asterisk refer to results shown to experts. (PDF) [file pone.0296241.s002.pdf]

S3 Table: Whale-watching experts' questionnaire. Questions between both questionnaires are linked by their identifier (ID). Questions marked with an asterisk refer to results shown to experts.

**INTERACTION BETWEEN SHIPS AND MARINE FAUNA**		**QUESTION ID**
Only 4% selected 'All of them'. The least selected options were biofouling and chemical pollution. Why do you think some of the options were not selected? Select all that might apply. (*)	• Low awareness of the impact<br>• Limited knowledge of the subject<br>• Both of the above<br>• I don't know	ID 3
Do you think that education and outreach activities for operators would increase their awareness of the potential impacts of whale-watching vessels?	• Yes<br>• No<br>• I don't know	
Do you think that education and outreach activities for operators would motivate them to reduce impacts of whale-watching vessels on marine fauna?	• Yes<br>• No<br>• I don't know	
**YOUR OPINION ON TOURISTS' PRIORITIES**		
What are the most important aspects for tourists during whale-watching trips? Select all that might apply.	• Going very close to the whales<br>• Being taught something about the biology/ecology of the whales<br>• Seeing as many animals as possible<br>• Getting to know something about the marine environment of the area<br>• Seeing at least one whale during the trip<br>• I don't know<br>• Other	ID 4
**TOURISTS' PRIORITIES ACCORDING TO WHALE-WATCHING OPERATORS**		

We expected that tourists' priority was to see or get close to whales. However, according to whale-watching operators, the two most important aspects for tourists are: (*)	• Getting to know something about the marine environment of the area<br>• Being taught something about the biology/ecology of the whales	
Do you think that education and outreach regarding tourism impacts on marine fauna would help operators manage tourists' expectations? (e.g., in case they do not encounter a whale)	• Yes<br>• No<br>• I don’t know	
