# Supplementary material for: The role of operators in sustainable whale-watching tourism: Proposing a continuous training framework
Source: PLoS One. 2024 Jan 2;19(1):e0296241. doi: 10.1371/journal.pone.0296241 (PMC10760867; doi:10.1371/journal.pone.0296241)
Supplement: S3 Table — Expert opinions on the most important aspects for tourists during whale-watching trips. The options selected by 2 out of 3 experts are shown in bold, while the options selected by one expert are shown in normal text. (PDF) [file pone.0296241.s003.pdf]

S6 Table: Expert opinions on the most important aspects for tourists during whale-watching trips. The options selected by 2 out of 3 experts are shown in bold, while the options selected by one expert are shown in normal text.

What are the most important aspects for tourists during whale-watching trips? Select all that might apply.
**Getting to know something about the marine environment of the area**
**Seeing at least one whale during the trip**
**Going very close to the whale**
**Seeing as many animals as possible**
Enjoying the camaraderie of watching wildlife in a good group
Getting out on the water
Feeling like they experienced something special
Being taught something about the biology/ecology of the whales
