# Supplementary material for: The role of operators in sustainable whale-watching tourism: Proposing a continuous training framework
Source: PLoS One. 2024 Jan 2;19(1):e0296241. doi: 10.1371/journal.pone.0296241 (PMC10760867; doi:10.1371/journal.pone.0296241)
Supplement: S1 Text — (PDF) [file pone.0296241.s004.pdf]

Whale-watching tour operators survey

Dear participant,
We are a group of researchers based in Canada, Italy, Sweden.

Through this questionnaire, we wish to know your opinion, as a tour operator, regarding different aspects related to whale-watching activities.

Participation in this research study is anonymous, and your responses will be treated confidentially.
You have the right to withdraw from the study at any time by simply closing the page.

Note: if you are currently off-season, your answers can reflect your last work season.

The questionnaire will take less than 5 minutes to be completed.
You do not need to sign in/up to google to complete the survey.
Please, remember to click on "submit" at the end of the questionnaire.

THANK YOU FOR TAKING THE TIME TO PARTICIPATE IN OUR RESEARCH!

Feedback is welcomed via email:
Alice Affatati, Memorial University, aaffatati@mun.ca;
Chiara Scaini, National Institute of Oceanography and Applied Geophysics, cscaini@inogs.it;
Anna Scaini, University of Stockholm, anna.scaini@natgeo.su.se.

* Required

1. Human beings rely on ecosystem services provided by the ocean. Human health and well-being depend on the availability of ecosystem service, which link human, animal health and the environment. Many scientific studies explore the connection between biodiversity and human health and the benefits that humans obtain from the ecosystem. However, regarding whale-watching, all the surveys published in scientific papers have dealt with what tourists expect from whale-watching tours. In this project we are looking at ecosystem conservation from a variety of points of view: marine ecosystems, citizen science, physical geography. Since whale-watching operators in Canada are a very responsible category, sensitive to conservation issues, we thought it would be interesting to know how much they think underwater noise can be considered an issue. We would like to gather your perspective on general underwater noise issues. If you agree to participate in the study, please tick the box here: *

Check all that apply.

☐ I consent to participating in the study described in the document

Introductory questions

Your answers will help us understand more about the type of vessels you use.

2. How long have you been working in the whale-watching industry? *

Mark only one oval.

- ( ) 1-5 years
- ( ) 5-10 years
- ( ) more than 10 years

3. How many boats do you use for your whale-watching tours? *

Mark only one oval.

- ( ) 1
- ( ) 2
- ( ) 3
- ( ) 4
- ( ) 5
- ( ) more than 5

4. What type of boats do you use? Select all that might apply. *

Check all that apply.

- [ ] Zodiac
- [ ] Sailboat
- [ ] Cruiser
- [ ] Other
- [ ] Fishing boat

5. Do your vessels have an Automatic Identification System (AIS)? *

Mark only one oval.

- Yes
- No
- Not applicable

Trip specific questions

Your answers will help us understand more about your work as a whale-watch operator.

6. How many tours per day do you do? *

Mark only one oval.

- 1
- 2
- 3
- more than 3

7. What time of day do you go out for your tours? Select all that might apply. *

Check all that apply.

- [ ] Mornings
- [ ] Afternoons
- [ ] Evenings

8. Which period of the year? Select all that might apply. *

Check all that apply.

- [ ] Winter (December-February)
- [ ] Spring (March-May)
- [ ] Summer (June-August)
- [ ] Fall (September-November)

9. Which routes do you usually take? Choose a number corresponding to the area on the map (Image modified from Google Earth). Select all that might apply. *

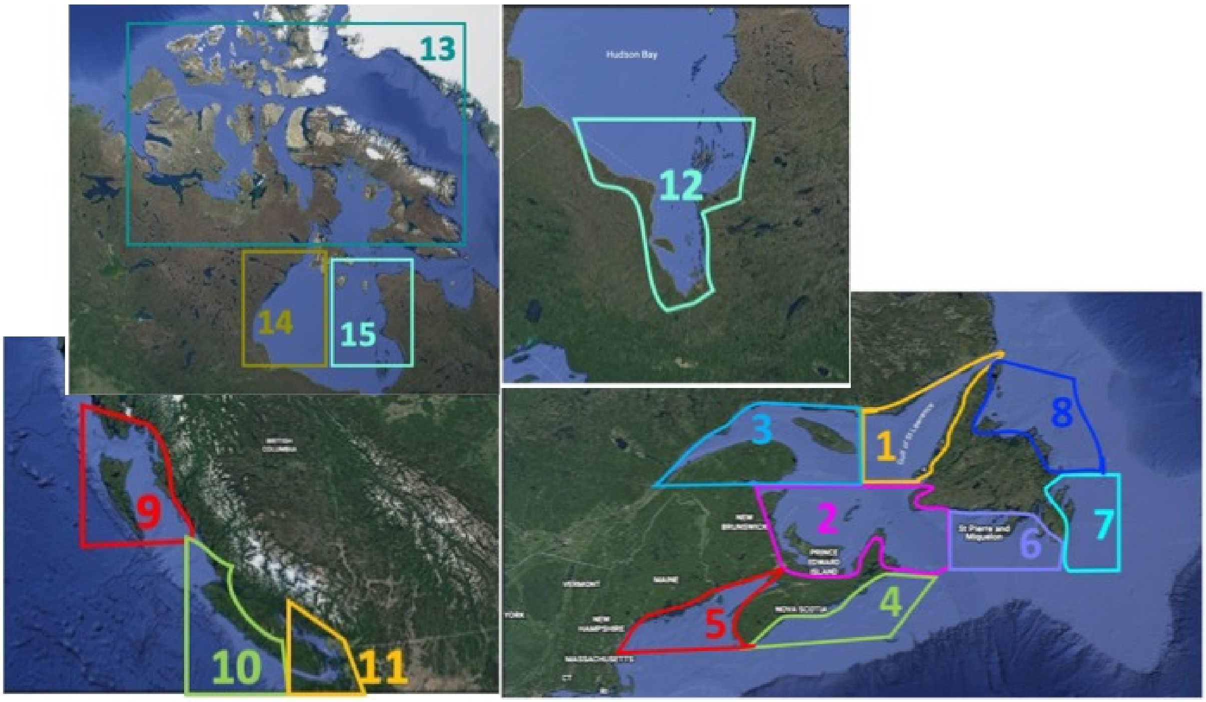

Check all that apply.

- [ ] 1
- [ ] 2
- [ ] 3
- [ ] 4
- [ ] 5
- [ ] 6
- [ ] 7
- [ ] 8
- [ ] 9
- [ ] 10
- [ ] 11
- [ ] 12
- [ ] 13
- [ ] 14
- [ ] 15

10. Do you change your routes? *

Mark only one oval.

- Yes
- No

11. Which cetaceans do you usually encounter? Select all that might apply. *

Check all that apply.

- [ ] Killer whales (Orcinus orca)
- [ ] Humpback whales (Megaptera novaeangliae)
- [ ] Fin whales (Balaenoptera physalus)
- [ ] Minke whales (Balaenoptera acutorostrata)
- [ ] Sperm whales (Physeter macrocephalus)
- [ ] North Atlantic Right Whales (Eubalaena glacialis)
- [ ] Beluga whales (Delphinapterus leucas)
- [ ] Other

12. Do you think these animals have increased or decreased in number after the start of the COVID-19 pandemic (and general decreased shipping traffic). *

Mark only one oval.

- Increased
- Decreased
- The number is more or less the same
- I don't know

13. How far away does your vessel go from the whale during the tours? *

Mark only one oval.

- more than 100 m
- more than 200 m
- less than 10 m
- 10-50 m
- around 100 m
- I don't know

14. Do you think that there are any issues related to the interaction between ships and marine fauna? Select all that might apply. *

Check all that apply.

- Chemical Pollution
- Ship strikes
- Noise
- Introduction of invasive species
- Biofouling
- I don't know
- None of them
- All of them

Underwater noise

Your answers will help us understand more about your relation with cetaceans as a whale-watching tour operator. In particular, we are interested in the acoustic interaction between whale-watching activity and cetaceans.

Schematic examples of various underwater acoustic sources (Image by Alice Affatati, art not to scale)

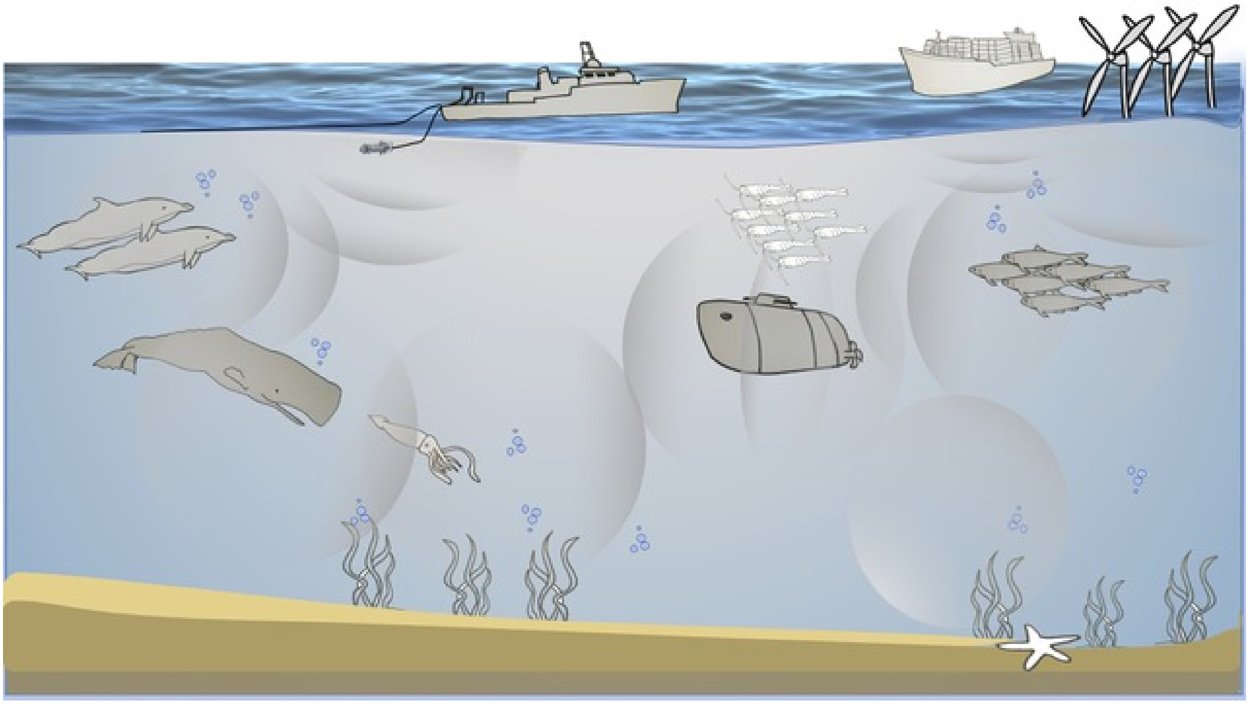

15. What are the most important aspects for tourists? Select all that might apply. *

Check all that apply.

- [ ] Going very close to the whales
- [ ] Being taught something about the biology/ecology of the whales
- [ ] Seeing as many animals as possible
- [ ] Getting to know something about the marine environment of the area
- [ ] Other
- [ ] Seeing at least one whale during the trip
- [ ] I don't know

16. Are you aware of the Fisheries and Oceans Canada (DFO) guidelines you should abide when encountering a cetacean? *

Mark only one oval.

- ( ) Yes
- ( ) No

17. How far away should the vessel be from the cetacean, at least? (Image by Alice Affatati, art not to scale) *

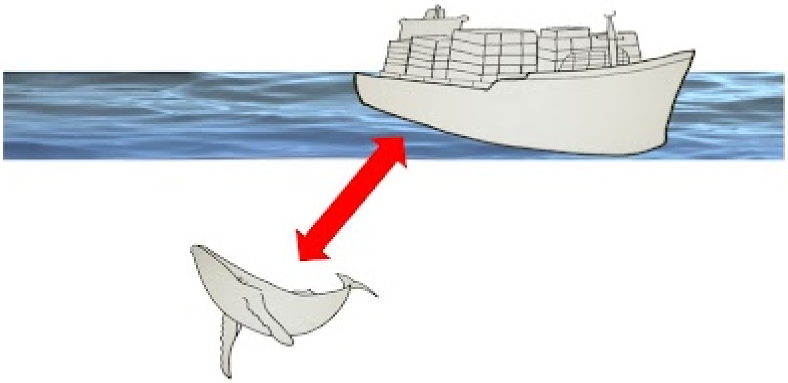

Mark only one oval.

- 100 m
- 200 m
- 300 m
- 350 m
- I don't know
- It doesn't matter

18. Do you think that underwater noise from shipping affects cetaceans more if they are travelling or feeding? *

Mark only one oval.

- Travelling
- Feeding
- I don't know
- Both equally

19. Which of these strategies would you prefer to follow in order to reduce the impact of underwater noise from shipping on cetaceans? (Select all that might apply) *

Check all that apply.

- [ ] Implement mandatory AIS on all vessels (even small recreational ones)
- [ ] Increase distance from the whales
- [ ] Decrease the speed of the vessel near whale migratory routes or selected areas
- [ ] Increase the number of Marine Protected Areas with limited access for vessels with a permit
- [ ] Implement mandatory avoidance of feeding and breeding areas during the most important times of day for these activities
- [ ] Diminish the duration of the whale-watching trips
- [ ] Avoid whale-watching tours during specific times of day
- [ ] Increase the duration of the whale-watching trips, but reduce the trips to one, daily

20. Select the species that are considered Endangered (select all that might apply) *

Check all that apply.

- [ ] North Atlantic right whale (Eubalaena glacialis)
- [ ] Humpback whale (Megaptera novaeangliae)
- [ ] Fin whale (Balaenoptera physalus)
- [ ] Blue whale (Balaenoptera musculus)
- [ ] I don't know
- [ ] None of them
- [ ] All of them

21. What other animals are also impacted by underwater noise? (Select all that might apply) *

Check all that apply.

- [ ] Fish
- [ ] Crustaceans
- [ ] Sea turtles
- [ ] Seabirds
- [ ] Walruses
- [ ] Manatees
- [ ] None of them
- [ ] All of them
- [ ] I don't know

THANK YOU FOR YOUR PARTICIPATION

Feel free to contact us if you want to know more about this research or if you are curious about the results!
Alice (aaffatati@mun.ca)
Chiara (cscaini@inogs.it)
Anna (anna.scaini@natgeo.su.se)

PLEASE, REMEMBER TO CLICK ON THE "SUBMIT" BOTTON BELOW, OTHERWISE YOUR RESPONSE WON'T BE RECORDED.

This content is neither created nor endorsed by Google.

Google Forms
