# Supplementary material for: The role of operators in sustainable whale-watching tourism: Proposing a continuous training framework
Source: PLoS One. 2024 Jan 2;19(1):e0296241. doi: 10.1371/journal.pone.0296241 (PMC10760867; doi:10.1371/journal.pone.0296241)
Supplement: S2 Text — (PDF) [file pone.0296241.s005.pdf]

Enhancing sustainable whale-watching tourism

Dear participant,
We are a group of researchers based in Italy and Sweden.

Through this questionnaire, we wish to know your opinion, as a whale-watching expert, regarding different aspects related to whale-watching activities and results we obtained, to a previous questionnaire, from whale-watching operators' responses.

Participation in this research study is voluntary and your expert opinion will be used in our publications. In the last section, you will be able to choose whether you decide to stay anonymous or be cited.
You have the right to withdraw from the study at any time by simply closing the page.

The questionnaire will take less than 5 minutes to be completed.
You do not need to sign in/up to google to complete the survey.
Please, remember to click on "submit" at the end of the questionnaire.

Feedback is welcome via email:
Alice Affatati, aliceaffatati@gmail.com;
Chiara Scaini, National Institute of Oceanography and Applied Geophysics, cscaini@inogs.it;
Anna Scaini, Stockholm University, anna.scaini@natgeo.su.se.

* Required

1. THANK YOU FOR TAKING THE TIME TO PARTICIPATE IN OUR RESEARCH! *

Check all that apply.

☐ I consent to participating in the study described above

INTERACTION BETWEEN SHIPS AND MARINE FAUNA

We asked whale-watching operators to select the issues related to the interaction between ships and marine fauna.

These are the results:

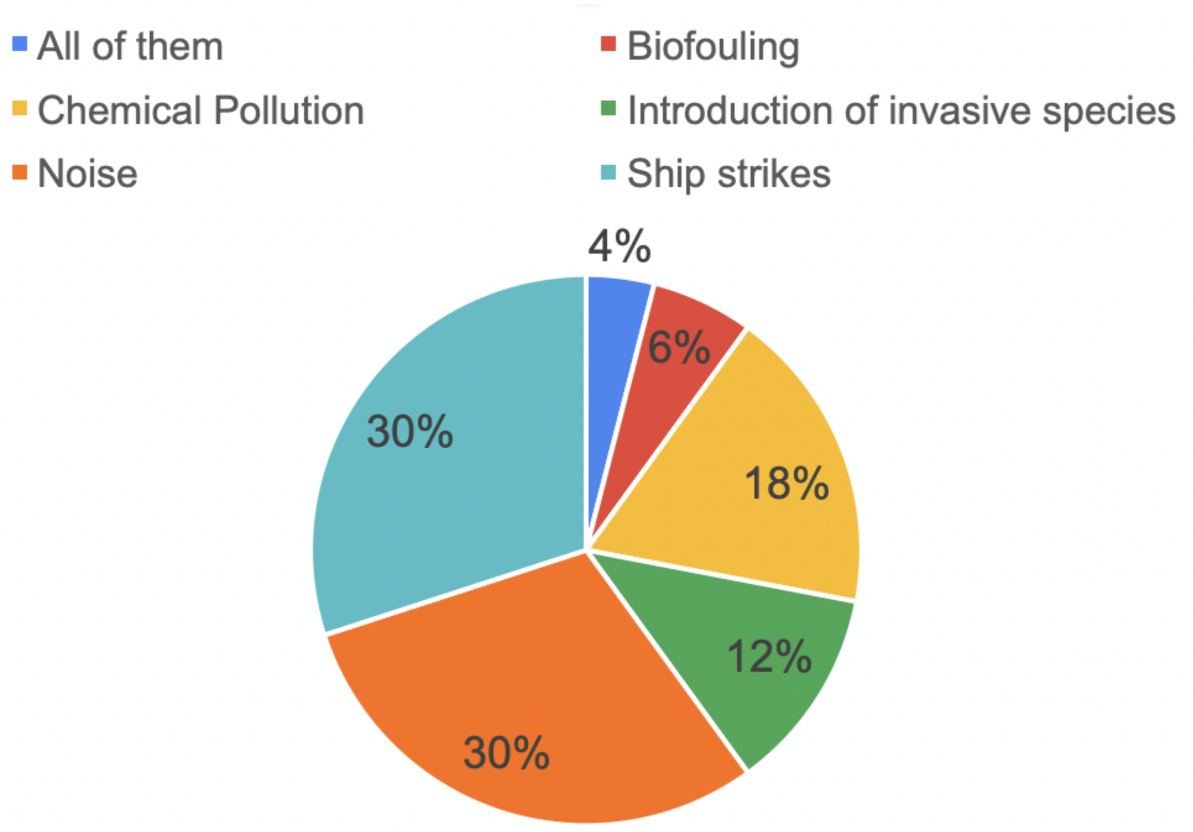

2. Only 4% selected 'All of them'. The least selected options were biofouling and chemical pollution. Why do you think some of the options were not selected? Select all that might apply. *

Check all that apply.

- [ ] Low awareness of the impact
- [ ] Limited knowledge of the subject
- [ ] Both of the above
- [ ] I don't know
- [ ] Other: ____________

3. Do you think that education and outreach activities for operators would increase their awareness of the potential impacts of whale-watching vessels? *

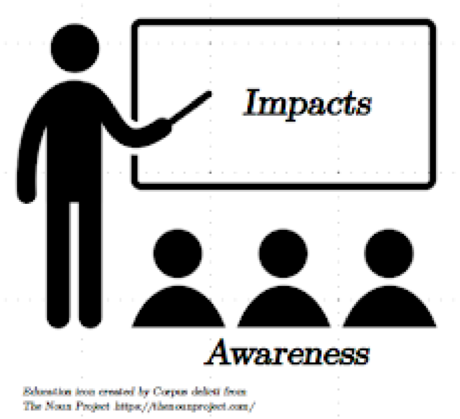

Mark only one oval.

- ◯ Yes
- ◯ No
- ◯ I don't know

4. Do you think that education and outreach activities for operators would motivate them to reduce impacts of whale-watching vessels on marine fauna? *

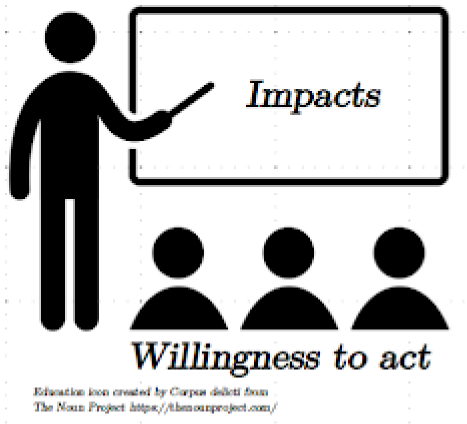

Mark only one oval.

- ◯ Yes
- ◯ No
- ◯ I don't know

5. Would you like to say something more?

____

____

____

____

____

YOUR OPINION ON TOURISTS' PRIORITIES

6. What are the most important aspects for tourists during whale-watching trips? Select all that might apply. *

Check all that apply.

- [ ] Going very close to the whales
- [ ] Being taught something about the biology/ecology of the whales
- [ ] Seeing as many animals as possible
- [ ] Getting to know something about the marine environment of the area
- [ ] Seeing at least one whale during the trip
- [ ] I don't know
- [ ] Other: _______________

TOURISTS' PRIORITIES ACCORDING TO WHALE-WATCHING OPERATORS

Tourists priorities during whale-watching trips according to whale-watching operators are:

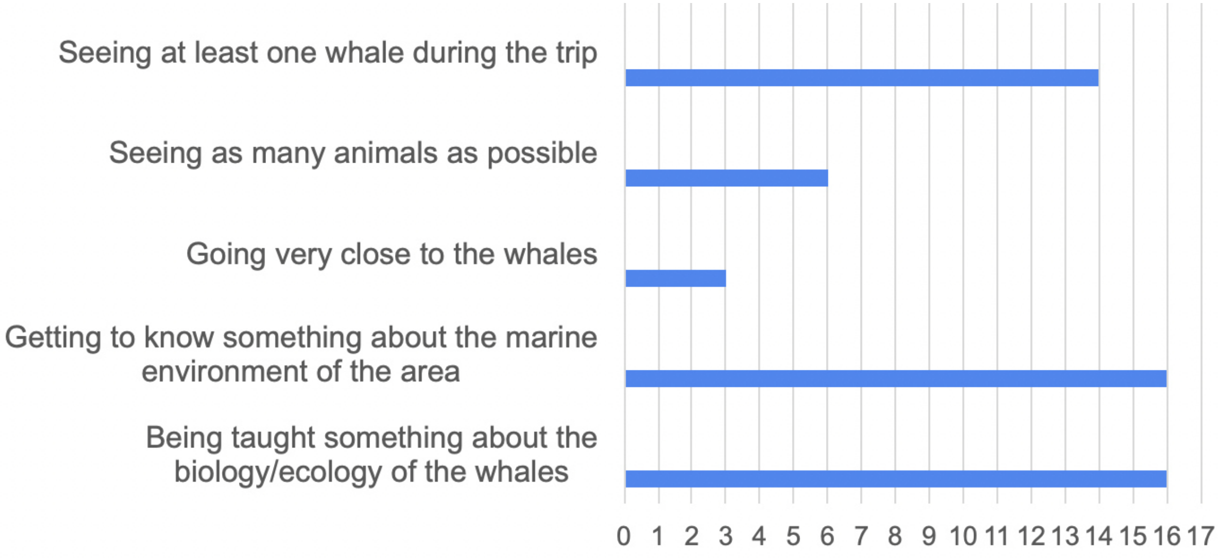

We expected that tourists' priority was to see or get close to whales. However, according to whale-watching operators, the two most important aspects for tourists are:

- Getting to know something about the marine environment of the area
- Being taught something about the biology/ecology of the whales

7. Do you think that education and outreach regarding tourism impacts on marine fauna would help operators managing tourists' expectations? (e.g., in case they do not encounter a whale) *

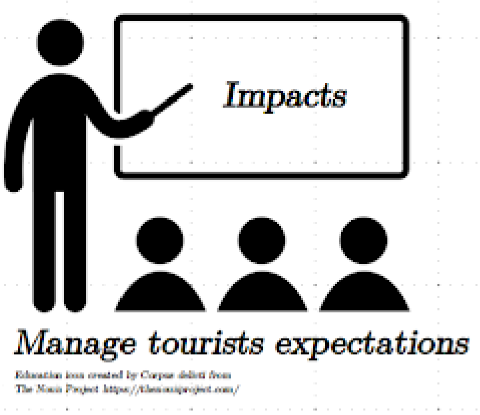

Mark only one oval.

- ◯ Yes
- ◯ No
- ◯ I don't know

8. Would you like to say something more?

____

____

____

____

____

THANK YOU FOR YOUR CONTRIBUTION	You experience in this field is very valuable to us, and we would like to cite you as a contributing expert in our paper upon your agreement. We will send you the manuscript draft for your approval.

9. I agree to be cited in the paper *

Mark only one oval.

- Yes
- No
- Other: ____________________

10. If you agreed, state your name:

____

11. Feel free to contact us if you want to know more about this research or if you are curious about the results! Alice (aliceaffatati@gmail.com), Chiara (cscaini@inogs.it), Anna (anna.scaini@natgeo.su.se). If you have any additional comment, please use the space below. PLEASE, REMEMBER TO CLICK ON THE "SUBMIT" BOTTON BELOW, OTHERWISE YOUR RESPONSE WON'T BE RECORDED.

This content is neither created nor endorsed by Google.

Google Forms
