# Supplementary material for: The role of operators in sustainable whale-watching tourism: Proposing a continuous training framework
Source: PLoS One. 2024 Jan 2;19(1):e0296241. doi: 10.1371/journal.pone.0296241 (PMC10760867; doi:10.1371/journal.pone.0296241)

S5 Figure: Main results from the whale-watching companies' website analysis. British Columbia (BC) is shown on the left column, Nova Scotia (NS) in the middle, and the whole dataset for Canada on the right. (A) Mention of impacts on marine fauna. In the "Canada" column, results for Nova Scotia (NS), Prince Edward Island (PE), and Nunavut (NU) are not displayed. (B) Mention of best practices. In the "Canada" column, results for Prince Edward Island (PE) and Nunavut (NU) are not displayed. (C) Mention of distance kept from mammals. In the "Canada" column, results for Manitoba (MB) and Quebec (QC) are not displayed. For the whole Figure: NL = Newfoundland and Labrador, BC = British Columbia, MB = Manitoba, NB = New Brunswick, QC = Quebec, MB = Manitoba and NS = Nova Scotia.

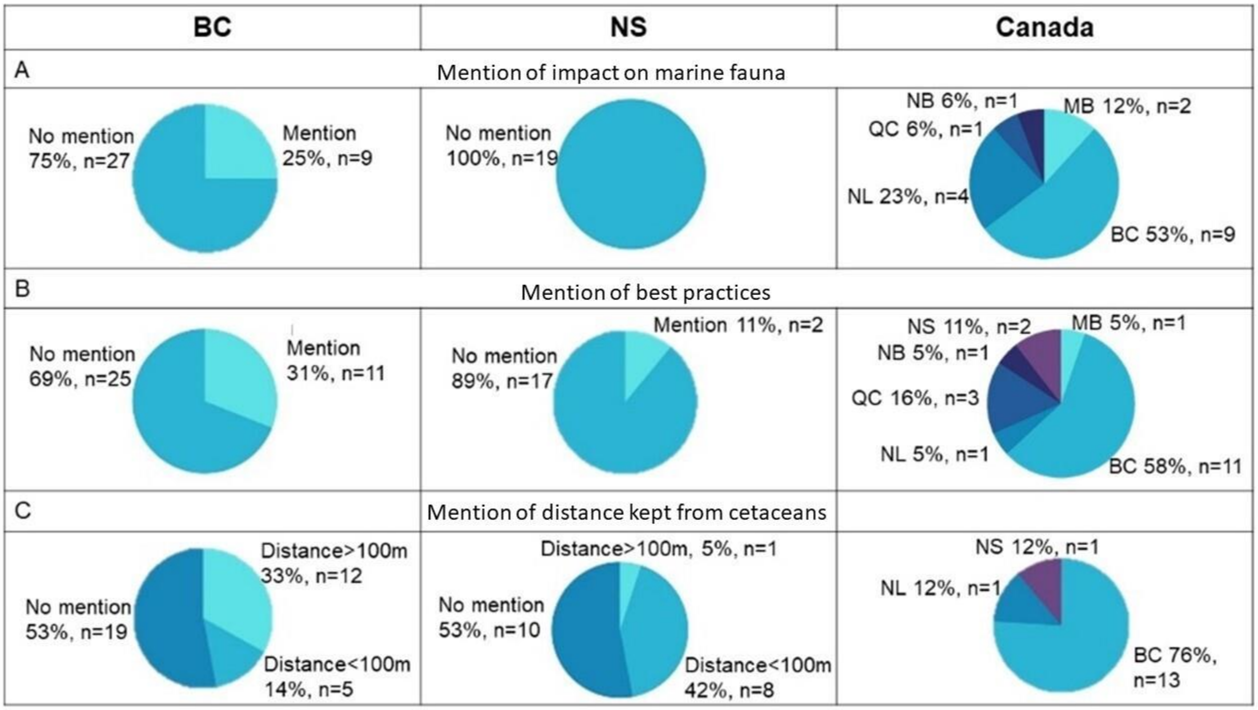

Supplement: S1 Fig — Main results from the whale-watching companies’ website analysis. British Columbia (BC) is shown on the left column, Nova Scotia (NS) in the middle, and the whole dataset for Canada on the right. (A) Mention of impacts on marine fauna. In the “Canada” column, results for Nova Scotia (NS), Prince Edward Island (PE), and Nunavut (NU) are not displayed. (B) Mention of best practices. In the “Canada” column, results for Prince Edward Island (PE) and Nunavut (NU) are not displayed. (C) Mention of distance kept from mammals. In the “Canada” column, results for Manitoba (MB) and Quebec (QC) are not displayed. For the whole Figure: NL = Newfoundland and Labrador, BC = British Columbia, MB = Manitoba, NB = New Brunswick, QC = Quebec, MB = Manitoba and NS = Nova Scotia. (PDF) [file pone.0296241.s006.pdf]
