# Supplementary material for: The role of operators in sustainable whale-watching tourism: Proposing a continuous training framework
Source: PLoS One. 2024 Jan 2;19(1):e0296241. doi: 10.1371/journal.pone.0296241 (PMC10760867; doi:10.1371/journal.pone.0296241)
Supplement: Attachment — Submitted filename: PONE-D-22-32212R1_Response to Reviewers.docx [file pone.0296241.s008.docx]

Academic Editor

PLOS ONE

Journal Requirements:

Please review your reference list to ensure that it is complete and correct. If you have cited papers that have been retracted, please include the rationale for doing so in the manuscript text, or remove these references and replace them with relevant current references. Any changes to the reference list should be mentioned in the rebuttal letter that accompanies your revised manuscript. If you need to cite a retracted article, indicate the article’s retracted status in the References list and also include a citation and full reference for the retraction notice.

*→ The reference list was reviewed and no reference was removed. Some minor corrections were undertaken thanks to the reviewer´s work and a careful check for typos.*

**Reviewer #1**

Thank you very much for your revised version of your manuscript and your detailed replies to my comments and criticism!

*→ Thank you for providing such great and constructive comments.*

“The research was set up during the COVID-19 pandemic lockdown. Therefore, online questionnaires were the only possible option.” => Really? What about Zoom interviews or phone calls? I personally know a lot of researchers using these approaches during the lockdowns… Anyway…

*→ Yes, we agree that questionnaires were not the only possible option, we could have presented this point more carefully. This response has been mentioned by Reviewer 3 as well. We have added some more details regarding the choice of the methodology in the main text, and amended this point.*

In Table 4 it must “Tourists’ preferences…”, so with a genitive form, thanks.

*→ We have incorporated this correction.*

In Reference 4 Line 540 there is a typo: “G$ssling” instead of “Gössling”, please correct.

*→ Thanks for checking the references, we have amended the typo.*

Reference 21: doi is missing.

*→ We have added the doi.*

All in all, I think the manuscript has been much improved and could be accepted now for publication.

**Reviewer #3**

The manuscript provides the perspective of Canadian whale-watching tour operators on responsible behaviour during the tours and their awareness of the potential impacts that the activity may cause. This view is complemented by the analysis of operators’ website contents and contrasted with the opinion of three experts. The study highlights the necessity of considering operators' perceptions to identify weaknesses and suggests a continuous training framework to address them. Considering that whale-watching is continuously increasing worldwide, and it has a large socio-economic impact in several countries, it is essential to find ways to understand how their practices can be responsibly improved and their potential impacts mitigated.

Overall the manuscript is well written and structured, with notable improvements after the first revision. The methodology is based mostly on online surveys, and one of the main constraints is the limited number of surveys considered (as previous reviewers have pointed out, and the authors have already argued in the text). However, the novelty of the study relies on the combination of approaches to address the same topic, and I particularly appreciate the creation of a simple index to objectively quantify the online communication strategies of the companies.

I suggest some minor revisions before publication to clarify some details in the text.

*→ We thank the reviewer for their work. Below we address each point and clarify the details as suggested. In addition, we have carefully revised the whole manuscript to improve the text.*

ABSTRACT

P19, L13. Following the style of the (original) title (positive!), I suggest rewriting this sentence to start with something positive too (e.g. "Whale watching is considered a form of green tourism"... "the threats you say etc demand a better understanding to develop a more sustainable industry").

*→ We have modified the sentence to give a more positive tone as suggested.*

INTRODUCTION

P20, L30. To start in a more assertive way (instead of having the “notwithstanding” among the first words). E.g. “Accompanying the rise of ecotourism”..

*→ We have modified the sentence accordingly. It now reads (lines 29-31):*

*“Tourist activities impact ecosystems directly, e.g., through habitat degradation, pollution, and loss of biodiversity, and indirectly by affecting ecosystem services provision, and despite the rise of ecotourism practices (refs)”.*

P20, L34. “…has been increasing worldwide…” since when? Add some temporal context.

*→ We have added “since the early ´90s” (as reported by the referenced literature).*

P20, L53. improve -> improving

P21, L56. Delete “and projections”

*→ We have modified the sentences at page 20 and 21 as suggested.*

METHODS

The advantages you cite in the response to Reviewer #1 (P96) are of interest to support your methodology choice. I would add them to the manuscript.

*→ We have improved the information to support the methodological choice in the main text as suggested (lines 331-332):*

*“Online questionnaires were chosen because of their time and cost efficiency, flexibility, lowered interviewer bias, and due to the unrestricted geographic coverage (Chang & Vowles, 2013)”.*

*Chang, T. Z. D., & Vowles, N. 2013. Strategies for improving data reliability for online surveys: A case study. International Journal of Electronic Commerce Studies, 4(1), 121-130.*

P27, L195. I only realised when reading the results what these columns refer to. I don't think the column indication is needed here. Clear enough (even clearer!) without it.

*→ The reference to the columns was deleted as suggested.*

P28, L215. The section you refer to should be section 2.1 (instead of 2.2.1).

*→ The section was corrected.*

RESULTS

P23, L119. Regarding ID 1, it should be good to briefly indicate why you used these distances, I would say based on current guidelines/legislation? Also check your reply to Reviewer#1 (P99 – Table 1, ID 1)

*→ The following text was added to clarify the choices (lines 138-143):*

*“We selected 100 and 200 m based on current guidelines and legislation. Canada's Marine Mammal Regulations mention “keeping a minimum of 100 meters away from most whales, dolphins, and porpoises, and keeping a minimum of 200 meters away if they are in resting position or with their calf.” Other reasonable distances in the upper and lower bound of the prescribed distance (displaying them in the question in an increasing order) were included in order to assess operators' knowledge, and to partially prevent biased responses”.*

P23, . Regarding ID 2, I think it would be also of interest to include the info of your response to Reviewer #1, P99 ID 2 about the increasing scale, as it helps to design further studies avoiding the same problem.

*→ A sentence was added to clarify this further (lines 144-145):*

*“We did not choose an increasing scale to try to avoid bias and discourage respondents towards selecting an intermediate number”.*

P29, L226-228. Do "companies", "operators" and "respondents" mean the same? it sounds a bit confusing here. I wonder if a company may have different operators, or if several respondents may come from the same company/operator? I suggest briefly clarifying this, or standardising the names for clarity.

*→ the text has been carefully reviewed to improve the wording by using the word “operators” instead of a mix of the three mentioned above.*

P29, FIG.1. Delete the names of the provinces where you don't have data to avoid noise in the image. In the caption, you should state the full name of the ones you use, i.e. BC, QC, NB, NS and NL (as you already did with BC and NS).

*→ Figure 1 was edited to delete the names of the provinces without data. The caption was also modified as suggested.*

P29, L239. “(The..)” -> (the..)

P30, L250. Is the same ONE company in both provinces? or one company in each of them? Please clarify.

*→ The sentence was modified to (line 255):*

*“Only one operator in BC and one in NS chose the correct option, “All of them””.*

P30, L252. “Most perceived issues have similar percentage…” In both areas?

*→ Yes, the sentence was modified to (lines 256-257):*

*“Most of the other issues had a similar percentage of responses in BC and NS, except for chemical pollution, which was indicated 8 times in BC and 1 time for NS.”*

P30, L262. “In NS waters, operators stated that they decrease the vessel’s speed (n=5) and implement mandatory avoidance of feeding and breeding areas”. But do they already comply with this? Or these are the options they prefer? Please, clarify.

*→ They state that they do so in the questionnaire. The sentence was modified as allows (lines 267-269):*

*“In NS waters, operators indicated in their responses that they decrease the vessel’s speed (n=5) and implement mandatory avoidance of feeding and breeding areas during the most important times of day for these activities (n=2)”.*

P31, L276. Delete (section 2.2). And if you already have added the name on the Fig. 1 caption, here you keep only the QC (as for BC). The same with the next 2 lines.

*→This is the first mention to Québec, so we kept the name. In the following lines we also have left the full names of the provinces, a+s this is the first time we mention them in the main text.*

P32, L284. As it refers to the same question, keep it in one sentence: “…general; in NS…”, and put the reference to the table for this, which should be Fig. S3A, instead of Fig S3.

*→ The sentence has been modified as suggested.*

P32, L285 and L289. Accordingly, Fig. S3B and Fig. S3C (at the end of the paragraph).

*→ The sentence has been modified as suggested.*

P32, L287. As it refers to the same question, keep it in one sentence: “…too close and 53%...”

*→ The sentence has been modified as suggested.*

P32, L293. Results from “operators” questionnaire…

*→ The sentence has been modified as suggested.*

P33, L307. I would state here the highlights of this answer. And keep the S4 as it is. Otherwise, this topic loses impact in the manuscript, as no info is provided about these results.

*→ To address this point, we have revised the paragraph and added a summary of the most important points raised by the experts. The sentence now reads (lines 308-311):*

*“The experts chose what they believed to be the most critical aspects for tourists during whale-watching trips among the provided options, and added additional aspects including “enjoying the camaraderie of watching wildlife in a good group”, “getting out on the water”, and “feeling like they experienced something special” (Table S4). “*

DISCUSSION

P33, L315. As you are now in the discussion, I suggest re-writing differently this sentence (as it is now, it sounds more like methods). As an example: "A Sustainable Communication Index is introduced, as a novel tool to objectively analyse the online whale watching communication strategies (section 4.2).”

*→ The sentence has been modified as suggested.*

P33, L317. “The questionnaire for whale watching operators can…”

*→ The sentence was amended as suggestd.*

P34, L340. “…Orams stated that 35% of whale-watching tourists…” From where? Worldwide?

*→ The sentence has been modified to include the spatial scale of the study (lines 344-345):*

*“In a study conducted in Moreton Island, Australia, 35% of whale-watching tourists were satisfied with the trip even without encountering whales [45]”.*

P35, L369. Indicate the province between brackets for the St. Lawrence estuary and Saguenay River.

*→ the province of Québec was indicated as suggested.*

P36, L380. Be Whale Wise (big letter). Can you explain briefly what this is, or what’s the main goal or achievement of these guidelines?

*→ We have capitalized the letters and added some more information about the Be Whale Wise guidelines (lines 383-386):*

*“The Be Whale Wise goal is to educate about codes of conduct and best practices around the whale-watching experience; such best practices were mentioned on many operators´ websites (with sections called “code of conduct”, Table 5)”.*

*→ We have modified the whole paragraph to explain more clearly in what way whale-watching practices could be more aware of whale´s disturbance.*

P36, L381. Please confirm the Table number, I would say that you want to refer to Table 5 instead of 2.

*→ We indeed refer to Table 5 and have amended it in the text.*

P36, L398-399. Incomplete sentence.

*→ The sentence has now been amended; the end of the paragraph now reads (lines 401-404):*

*“However, younger tourists (for instance in [69] the average age was 38 years) might be more comfortable looking for information online. Not all tourists looking for a whale-watching tour will look for options online, and small companies might advertise their activities only on the docks”.*

P37, L412. “Analyzing […] was not possible…” Briefly explain why

*→ To explain this point, we have added to the sentence “because anonymity was kept for all participants” (lines 416-417).*

P37, L423-424. Delete or re-write “and the importance of managing passenger expectations [46]”.

*→ The last part of the sentence was deleted as suggested.*

P38, L436-441. I suggest explaining the Orams model before you propose modifications (i.e. L436).

*→ This part was modified following this suggestion. The text presenting the modified Orams model was sharpened (lines 441-450):*

*“The framework of the Orams model [45], initially developed to model tourists’ education, was here adjusted for whale-watching operators’ education, participatory and active training. We modified the Orams model by introducing a step in the training program denominated “Impacts awareness” (blue box, Fig. 2) as part of “The Affective Domain” and “Curiosity.”*

SUPPORTING INFORMATION

S5 and S6 are cited in the text before other S figures. Please consider re-numbering for consistency and organization.

*→ Following the reviewer’s suggestion, the supporting texts, figures, and tables have been renumbered following the order of appearance in the manuscript.*

P2, L16. Whale-watching operators questionnaire. (We already assume that a questionnaire has questions and that the table should be below).

*→ Thank you for pointing this out. We re-worded accordingly. Now the caption reads: “S1 Table: Questionnaire for whale-watching operators used in this paper”*

P4, L21. “Each question…” This sentence is not correct. Fix or maybe simplify like: Questions between both questionnaires are linked by their identifier (ID).

*→ We followed the reviewer’s suggestion and rewrote the sentence as “Questions between both questionnaires are linked by their identifier (ID).”*

P6, L26-32. As you have some common aspects for all the sections of the figure, please consider writing these first and indicate where these results come from: “Main results from the whale-watching companies website analysis. British Columbia (BC) on the left column, Nova Scotia (NS) in the middle and the whole dataset for Canada on the right. (A) Mention of impacts on marine fauna. Results for Nova Scotia (NS), Prince Edward Island (PE), and Nunavut (NU) are not displayed. (B) Mention of best practices. Results for Prince Edward Island (PE) and Nunavut (NU) are not displayed. (C) Mention of distance kept from mammals. Results for Manitoba (MB) and Quebec (QC) are not displayed. For the whole Figure: NL = Newfoundland and Labrador, BC = British Columbia, MB = Manitoba, NB = New Brunswick, QC = Quebec, MB = Manitoba and NS = Nova Scotia.”

*→ Thank you for suggesting this change. The new caption reads:*

*“Main results from the whale-watching companies’ website analysis. British Columbia (BC) is shown on the left column, Nova Scotia (NS) in the middle, and the whole dataset for Canada on the right. (A) Mention of impacts on marine fauna. In the “Canada” column, results for Nova Scotia (NS), Prince Edward Island (PE), and Nunavut (NU) are not displayed. (B) Mention of best practices. In the “Canada” column, results for Prince Edward Island (PE) and Nunavut (NU) are not displayed. (C) Mention of distance kept from mammals. In the “Canada” column, results for Manitoba (MB) and Quebec (QC) are not displayed. For the whole Figure: NL = Newfoundland and Labrador, BC = British Columbia, MB = Manitoba, NB = New Brunswick, QC = Quebec, MB = Manitoba and NS = Nova Scotia”.*
